# Upregulation of PIR gene expression induced by human papillomavirus E6 and E7 in epithelial oral and cervical cells

**DOI:** 10.1098/rsob.170111

**Published:** 2017-11-08

**Authors:** Diego Carrillo, Juan P. Muñoz, Hernán Huerta, Gabriel Leal, Alejandro Corvalán, Oscar León, Gloria M. Calaf, Ulises Urzúa, Enrique Boccardo, Julio C. Tapia, Francisco Aguayo

**Affiliations:** 1 Department of Basic and Clinical Oncology, Faculty of Medicine, University of Chile, Independencia 1027, PO 8389100, Santiago, Chile; 2 Advanced Center for Chronic Diseases (ACCDiS), Pontificia Universidad Católica de Chile, Santiago, Chile; 3 UC-Center for Investigational Oncology (CITO), Pontificia Universidad Católica de Chile, Santiago, Chile; 4 Virology Program, Instituto de Ciencias Biomédicas (ICBM), Faculty of Medicine, University of Chile, Santiago, Chile; 5 Center for Advanced Research, Tarapaca University, Arica, Chile; 6 Center for Radiological Research, Columbia University Medical Center, New York, NY, USA; 7 Department of Microbiology, Institute of Biomedical Sciences, University of Sao Paulo, Sao Paulo, Brazil

**Keywords:** cancer, papillomavirus, PIR, oral, cervical

## Abstract

The hallmark of high-risk human papillomavirus (HR-HPV)-related carcinogenesis is E6 and E7 oncogene overexpression. The aim of this work was to characterize epithelial oral and cervical cancer cells that express HR-HPV E6 and E7 oncoproteins. Transcriptomic assay using DNA microarrays revealed that PIR gene expression was detected in oral cells in an HR-HPV E6/E7-dependent manner. In addition, PIR was overexpressed in HPV-positive SiHa and Ca Ski cells, whereas it was undetectable in HPV-negative C33A cells. The PIR expression was dependent on functional HR-HPV E6 and E7 oncoproteins even though the E7 oncoprotein had higher activity to induce PIR overexpression in comparison with E6. In addition, using an siRNA for PIR silencing in oral cells ectopically expressing HR-HPV E6/E7, there was a significant increase in E-cadherin transcripts and a decrease in Vimentin, Slug, Zeb and Snail transcripts, suggesting that HR-HPV-induced PIR overexpression is involved in epithelial–mesenchymal transition. Furthermore, migration of PIR-silenced cells was significantly decreased. Finally, using inhibitors of some specific pathways, it was found that EGFR/ERK and PI3 K/AKT signalling pathways are important for E7-mediated PIR overexpression. It can be concluded that PIR gene expression is highly dependent on the expression of HR-HPV oncoproteins and is important for EMT regulation.

## 1. Background

Human papillomavirus (HPV) is a non-enveloped, epitheliotropic, double-stranded DNA virus with icosahedral symmetry. More than 200 HPV genotypes have been found and sequenced. Among them, high-risk HPV (HR-HPV) is a group of HPV genotypes (16, 18, 31, 33, 35, 45, 52 and 58, among others) that are the causal agents of cervical carcinomas. Furthermore, infection with these viruses has been associated with a significant proportion of anogenital and head and neck carcinomas (HNCs), including oropharyngeal and oral carcinomas [1,2].

Even though the mere overexpression of HR-HPV E6 and HR-HPV E7 oncoproteins is not a sufficient condition for cancer development, the hallmark of HR-HPV-mediated oncogenicity is E6 and E7 overexpression, which is generally caused by HR-HPV integration into the host genome [3]. HR-HPV E6 is a 160-amino-acid protein with two zinc-finger domains, and E7 is a 98-amino-acid protein that shows three conserved regions and only one zinc-finger domain. When HR-HPV-related cancers arise, the tumour cells become addicted to E6 and E7 oncoproteins, because when these oncoproteins are silenced, cells suffer senescence and apoptosis [4,5]. The oncogenic mechanism mediated by HR-HPV E6 and E7 oncoproteins is related to the ability of the virus to induce p53 and pRb destabilization with subsequent loss of apoptosis ability and cell cycle control, respectively [6]. In addition, HR-HPV E6 and E7 oncoproteins interact with many other proteins within epithelial cells for additional alterations [7]. Additional E6 partners include transcriptional co-activators, PSD95/Dlg/ZO-1 (PDZ) domain proteins, tumour suppressor proteins and inducers of apoptosis and factors related to DNA replication and repair [8]. It has been reported that the HR-HPV E6 oncoprotein induces the expression and activity of hTERT, the catalytic subunit of telomerase, which is assumed to be responsible for HR-HPV-mediated immortalization. Also, HR-HPV E7 binding to pRb family proteins induces E2F release and overexpression of the cyclin-dependent kinase inhibitor p16. Additional E7 partners are p21 [9], MPP2 [10], p48 [11] and p27 [12], among others. Thus, the overall ability of HR-HPV E6 and E7 for cell immortalization *in vitro* and cancer induction *in vivo* involves a complex network of interactions.

Even though there is increased knowledge on the alterations caused by HR-HPV interactions in epithelial cells, more information is required concerning molecular changes in HR-HPV associated with oral carcinogenesis when compared with cervical cancer. In this study, we found that pirin, the product of the PIR gene and an oxidative stress sensor, is consistently overexpressed in HR-HPV-associated tumours including head and neck and cervical cancer cells. Moreover, although both HR-HPV E6 and E7 oncoproteins are associated with PIR overexpression, E7 has a predominant effect in PIR expression. Finally, we found that HR-HPV-associated PIR overexpression is related to increased migration and epithelial–mesenchymal transition (EMT) properties of oral and cervical cancer cells expressing HR-HPV E6 and E7.

## 2. Results

### 2.1. Non-tumour oral epithelial cells express functionally active HPV16 E6 and E7 oncoproteins

To get new insights into the role of HPV-16 E6 and E7 oncogenes in epithelial oral cells, OKF6–Tert2 cells were stably transfected with a vector expressing HPV-16 E6 and E7 oncoproteins. Although this vector was designed for retroviral transduction by using appropriate packaging cells, previously published findings demonstrated that transfection is useful for efficient plasmid uptake [13,14]. Geneticin-resistant colonies were selected, pooled and characterized for expression of HPV oncoproteins and associated cellular alterations. These transfected cells, named OKF6HPV-16E6E7, were evaluated for E6 and E7 transcripts and oncoprotein expression using RT–qPCR and immunofluorescence (IF), respectively. As expected, these cells efficiently expressed HPV-16 E6/E7 transcripts and protein (as shown in electronic supplementary material, figure S1*a,b*). Most importantly, HPV-16 E6 and E7 oncoproteins were biologically functional because it caused p53 and pRb degradation (as shown in electronic supplementary material, figure S1*c*). Moreover, we observed that telomerase catalytic subunit (hTERT) transcripts were significantly increased (electronic supplementary material, figure S1*d*), supporting the biological functionality of E6 and E7 oncoproteins in OKF6–Tert2 cells. In addition, it was demonstrated that oral cells expressing HPV-16 E6/E7- exhibited an increased proliferation rate compared to parental cells or cells transfected with an empty vector (electronic supplementary material, figure S2).

### 2.2. PIR gene is overexpressed in oral epithelial cells expressing HR-HPV E6/E7 in a dose-dependent manner

**Figure 1.**
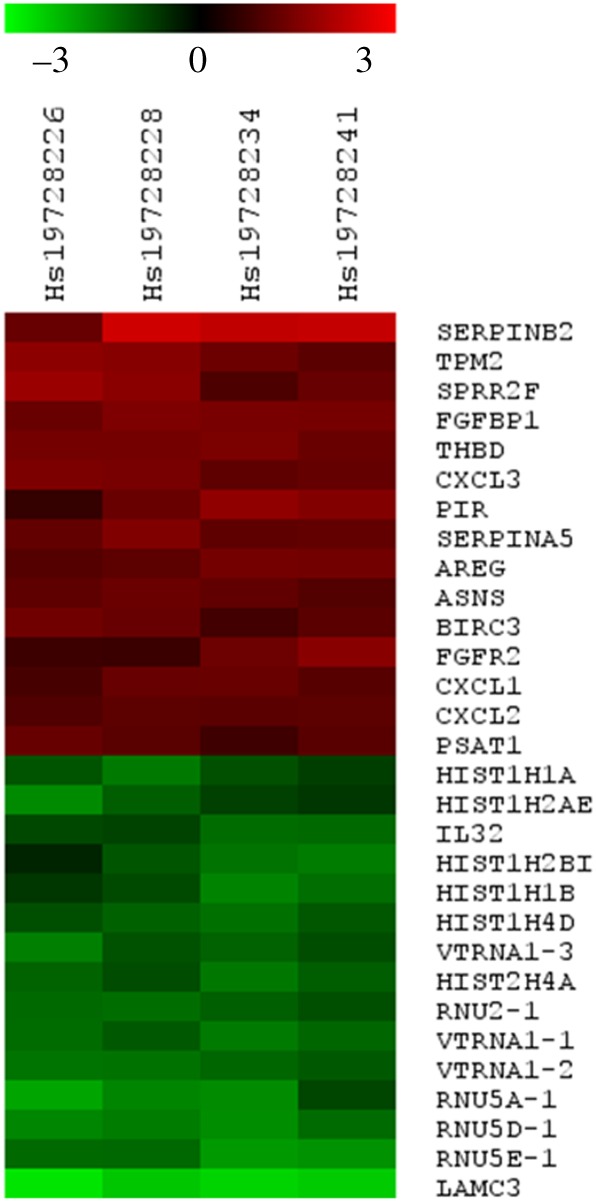
Heat map for up- and downregulated genes in OKF6HPV-16E6E7 cells. Total RNAs extracted from stably transfected (pLXSNHPV16E6E7) and empty vector transfected (pLXSN) OKF6–Tert2 cells were separately labelled by reverse transcription with Cyanine-5 and Cyanine-3 dCTPs dyes and co-hybridized to HEEBO-48 K microarrays. Raw data were collected as GPR files and normalized by print-tip Lowess. A LIMMA test was applied to determine differentially expressed genes using an adj-*p* value < 0.05 and a log2 fold-change > (±) 1.0 as cut-off. The 30 top-ranked up- and downregulated transcripts are shown.

**Figure 2.**
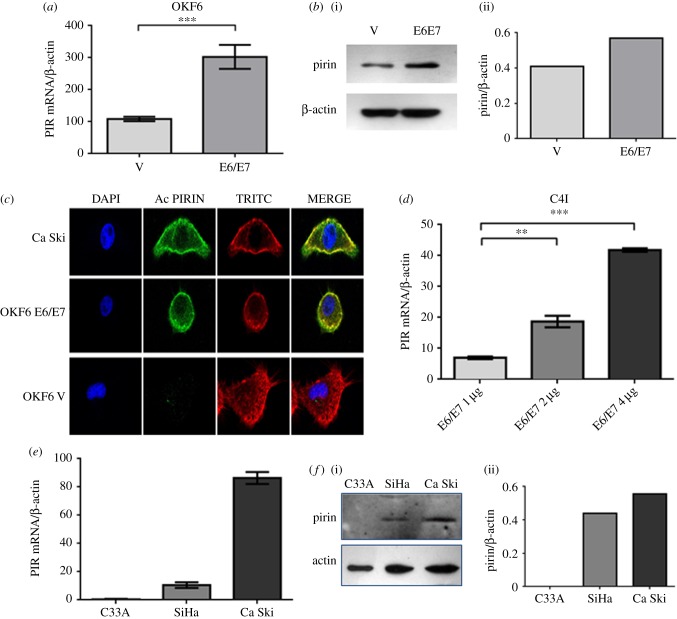
PIR transcripts and protein are overexpressed after ectopic expression of HPV-16 E6/E7 oncogenes in oral epithelial cells and in HR-HPV-positive cervix–uterine cancer cells. (*a*) PIR mRNA in OKF6-Tert 2 cells stably transfected with and empty vector and pLXSNHPV16E6E7 vector. (*b*)(i) Western blot for pirin detection in stably transfected OKF6–Tert2 cells; (ii) analysis by densitometry. (*c*) IF by confocal microscopy for pirin in OKF6–Tert2 cells stably transfected with pLXSNHPV16E6E7 or empty vector and Ca Ski cells. (*d*) C4I cervical cells were transiently transfected with different concentrations of pLXSNHPV16E6E7 vector, harvested after 24 h (80% confluence) and PIR transcripts were measured by RT–qPCR. (*e*) Cervical cancer cells (C33A, SiHa and Ca Ski) that contains 0, 2 and 500 copies of HPV-16 were analysed for PIR transcripts expression using RT–qPCR. (*f*) (i) Western blot for C33A, SiHa and Ca Ski protein extracts using antibodies for pirin and B-actin; (ii) densitometric analysis of western blot bands. Experiments were repeated at least three times with similar results. These results are representative of three independent experiments. **p < 0.01; ***p < 0.001.

In order to determine changes in the levels of mRNAs in OKF6HPV-16E6E7 cells, a transcriptomic approach with DNA microarrays was used (HEEBO, 48.5 K), by which these cells were tested for up- and downregulated genes. It was found that 37 genes were significantly upregulated and 85 genes were downregulated. A heatmap with the most important altered genes is shown in figure 1. We observed that PIR transcripts were strongly upregulated in these transfected cells (score: 2.4). It is known that the PIR gene encodes for pirin, a protein that was reported to be an oxidative stress sensor [15]. In fact, pirin is upregulated under tobacco smoke exposition in bronchial cells, and to our knowledge, no previous information has been reported about the relationship between this protein and HPV infection. Therefore, we focused on this protein for subsequent analysis. We confirmed the overexpression of PIR transcripts and pirin protein in OKF6HPV-16E6E7 cells using RT–qPCR and western blotting, as shown in figure 2*a,b*. In addition, the overexpression of pirin at protein level was confirmed in Ca Ski and OKF6HPV-16E6E7 cells by using indirect immunofluorescence (IFI). It was observed that Ca Ski cells, that harbour 500 HPV-16 copies per genome, expressed high levels of pirin (figure 2*c*). Next, a dose–response effect was analysed using C4I cervical cancer cells, which are HPV-18 positive although with low levels of pirin expression. These cells were transiently transfected with different concentrations of pLXSNHPV16E6E7 plasmids. A dose–response effect was observed using vector concentrations ranging from 1 to 4 µg ml^−1^ (figure 2*d*). Thus, we determined viral load dependency in HR-HPV-positive and -negative cervical cancer cells currently available in the laboratory. As expected, it was observed that PIR was overexpressed in a viral load-dependent manner at RNA and protein level in SiHa and Ca Ski cells, harbouring 2 and 500 HPV-16 copies/cell, respectively. However, C33A, an HPV-negative cervical cancer cell line, exhibited almost undetectable levels of pirin (figure 2*e*,*f*). Taken together, these results clearly showed that pirin overexpression is at least dependent on ectopic or endogenous HPV-16 E6 and E7 expression in oral and cervical cancer cells.

### 2.3. HPV-16 E7 oncoprotein is important for inducing PIR overexpression in oral and cervical cancer epithelial cells

**Figure 3.**
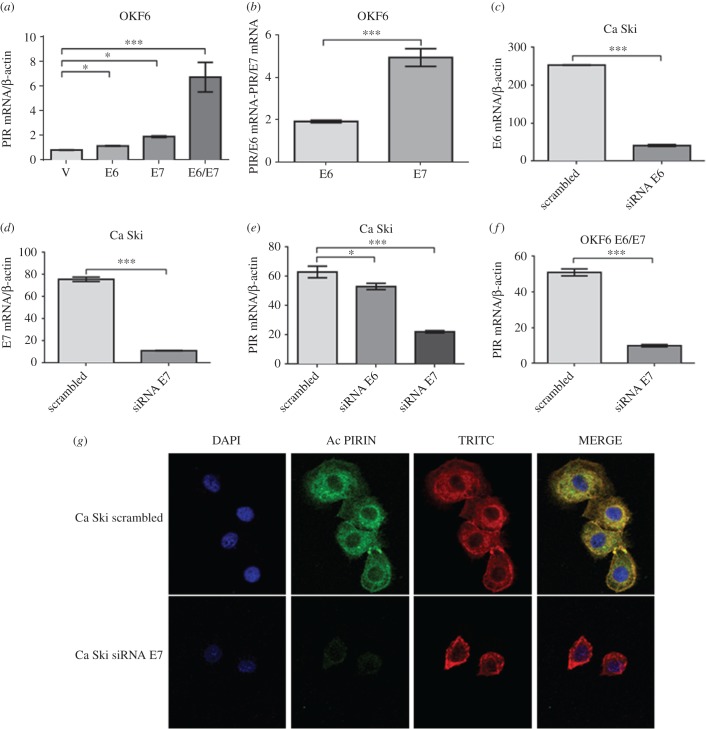
HPV-16 E7 activity is important for inducing PIR upregulation in tumour epithelial cells. (*a*) PIR mRNA levels in OKF6–Tert2 cells stably transfected with an empty pLXSN, pLXSNHPV16E6, pLXSNHPV16E7 or pLXSNHPV16E6/E7 vector. (*b*) PIR mRNA levels normalized against number of copies of HPV16 E6 or E7 transcripts. (*c,d*) HPV16 E6 and E7 mRNA levels in Ca Ski cells transiently transfected with an siRNA for E6 and E7 silencing, respectively. (*e*) PIR mRNA levels in Ca Ski cells that were transiently transfected with siRNA against HPV-16 E6, E7 or scrambled. (*f*) PIR mRNA levels in OKF6–Tert2 cells ectopically and stably expressing HPV-16 E6 and E7 oncoproteins and transiently transfected with a siRNA against E7. (*g*) IF for Ca Ski cells transiently transfected with a siRNA for E7 silencing. These results are representative of three independent experiments. *p < 0.05; ***p < 0.001.

In order to identify the HR-HPV oncoprotein involved in PIR overexpression, OKF6–Tert2 cells were independently transfected with pLXSNHPV-16E6 or pLXSNHPV-16E7 plasmids. After G418 selection, the corresponding pooled colonies were assayed for E6 or E7 transcript expression and protein functionality. Both HPV-16 E6 and E7 transfected OKF6–Tert2 cells efficiently expressed the corresponding transcripts and translated oncoproteins were functional, as demonstrated by p53 and pRb downregulation (electronic supplementary material, figure S3). Next, OKF6–Tert2 oral cells stably and ectopically expressing HPV-16 E6 or E7 oncogenes were analysed for PIR expression using RT–qPCR. As shown in figure 3*a*, both HPV-16 E6 and E7 oncoproteins independently induced PIR transcript expression, although E7 showed the higher activity. In addition, a synergism was apparent because PIR expression was significantly higher when both oncoproteins were expressed. However, when levels of PIR transcripts were normalized against levels of E6 or E7 transcripts, we observed that E7 is more efficient than E6 in inducing PIR expression, as shown in figure 3*b*. To confirm the importance of viral oncoproteins for PIR overexpression, an siRNA for HPV-16 E6 or E7 silencing was used. The results presented in figure 3*c*,*d* show that both siRNAs were able to silence E6 and E7 transcripts efficiently in Ca Ski cells. We confirmed that these siRNAs can silence E6 and E7 oncogenes in OKF6–Tert2 cells at RNA (electronic supplementary material, figure S3*a,b*) and protein levels (electronic supplementary material, figure S3*c*). In fact, both siRNA for HPV-16 E6 and E7 were able to decrease the levels of PIR transcripts in Ca Ski cells, although a higher decrease was observed when an siRNA against E7 was used compared to cells transfected with a scrambled siRNA (figure 3*e*). We confirmed the effect of siRNA against E7 using OKF6–Tert2 cells ectopically expressing E6 and E7 oncoproteins (figure 3*f*). Finally, it was found by IF that pirin protein is efficiently downregulated by transfection of Ca Ski cells with an siRNA for silencing HPV-16 E7 (figure 3*g*). We conclude that even though a synergism between HR-HPV E6 and E7 oncoproteins was observed, E7 oncoprotein is more effective than E6 for PIR upregulation.

### 2.4. PIR overexpression mediated by HPV-16 E7 is dependent on EGFR/MEK/ERK and PI3 K/Akt signalling pathways

**Figure 4.**
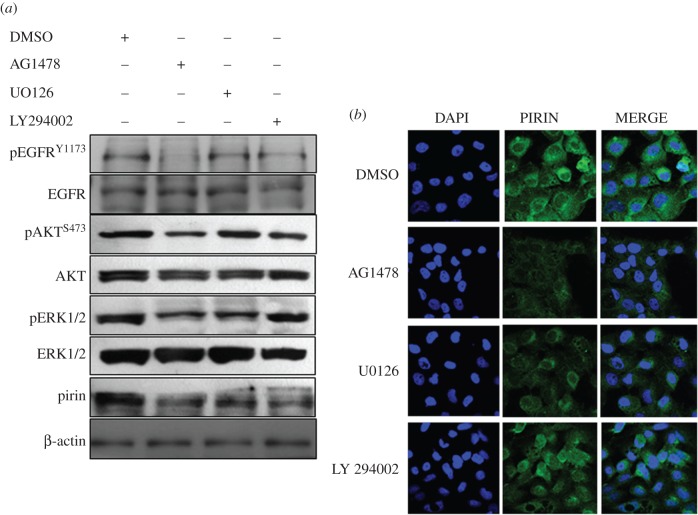
EGFR/MEK/ERK signalling pathway and PI3 K/AKT are involved in E7-mediated PIR overexpression. OKF6–Tert2 cells stably transfected with pLXSNHPV-16E7 vector were incubated with inhibitors AG1478 (EGFR tyrosine kinase), UO126 (MEK1/MEK2) and LY294002 (phosphoinositide 3-kinase). Pirin expression was evaluated by (*a*) western blot and (*b*) IF. Results are representative of three independent experiments.

To characterize the importance of mitogen-activated protein kinase (MAPK) and phosphoinositide 3-kinase (PI3 K) signalling pathways, potentially involved in HPV-mediated PIR overexpression, OKF6–Tert2 cells were stably transfected with pLXSNHPV-16E7 plasmid. Cells were synchronized by serum starvation during 24 h and incubated with the inhibitors AG1478 (EGFR tyrosine kinase), LY294002 (phosphoinositide 3-kinase) and UO126 (MEK1/MEK2). Both AG1478 and UO126 efficiently abolished pirin overexpression as observed in figure 4*a* (western blot) and figure 4*b* (IF). A decrease in pirin levels was observed with all of the inhibitors. However, in OKF6–Tert2 cells stably transfected with the empty vector, we did not observe significant changes in the levels of pirin protein after treatment with the inhibitors (electronic supplementary material, figure S6). These findings strongly suggest that EGFR/MEK/ERK and PI3 K/AKT pathways could be involved in the increase in pirin levels by HPV-16 E6 and E7 oncoproteins in oral cells.

### 2.5. Pirin is involved in epithelial–mesenchymal transition and migration in cells expressing HPV-16 E6/E7 oncogenes

**Figure 5.**
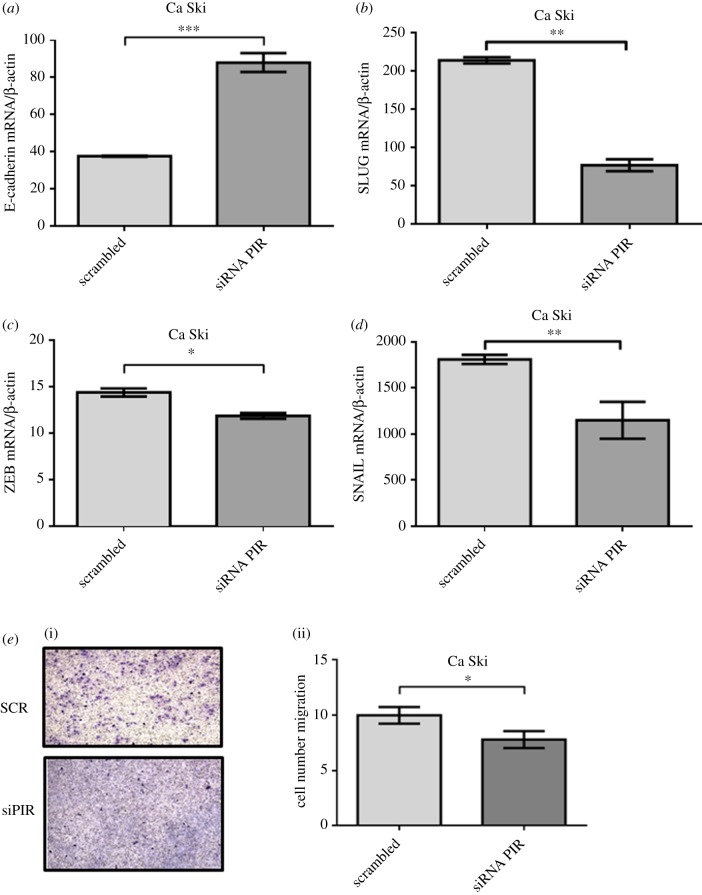
PIR expression is associated with increased EMT and migration in Ca Ski cells. (*a–d*) Ca Ski cells were transiently transfected with an siRNA for PIR silencing, and the mRNA levels of (*a*) E-cadherin, (*b*) SLUG, (*c*) ZEB and (*d*) Snail were measured by RT–qPCR. (*e*) (i) Transwell assay for Ca Ski migration after transfection with an siRNA for PIR silencing. (ii) Cell count after 7 h of migration. Results are representative of three independent experiments. *p < 0.05; **p < 0.01; ***p < 0.001.

In order to evaluate the role of pirin in tumours associated with HPV, we determined the levels of biomarkers related to EMT using a specific siRNA for PIR silencing. The efficiency of this siRNA to induce PIR silencing was determined in Ca Ski cells at RNA and protein levels (electronic supplementary material, figure S5*a*–*c*). We found that PIR silencing was associated with a significant increase in E-cadherin transcripts (*p* < 0.001) (figure 5*a*), and decrease in vimentin (*p* < 0.05), SLUG (*p* < 0.005), ZEB (*p* < 0.05) and Snail (*p* < 0.005) transcripts in Ca Ski cells (figure 5*b–e*), suggesting an important role of pirin in EMT regulation. In addition, using transwell migration assays, it was observed a significant decrease in the migratory ability of Ca Ski cells after incubation with an siRNA against PIR transcripts (figure 5*f*). These results were confirmed using SiHa cells (electronic supplementary material, figure S7). Taken together, these data strongly suggest that pirin is involved in EMT and migration in HPV-transformed cell lines.

## 3. Discussion

HR-HPV E6 and E7 oncoproteins are consistently overexpressed in all HR-HPV types of tumours frequently associated with HR-HPV integration into the host genome [16]. Even though the main molecular mechanisms, such as p53 and pRb destabilization, are hallmarks of HR-HPV-mediated carcinogenesis, more information is required about specific alterations in oral and cervical cancer cells and their relationship with HR-HPV. In this study, using a human whole-genome transcriptomic approach and confirmation by RT–qPCR, it was found that PIR was expressed at high levels in oral cells upon ectopic HPV-16 E6 and E7 oncoprotein expression. Also, we showed that this gene was strongly induced in HR-HPV-positive cervical cancer cell lines. However, PIR upregulation is not observed in HR-HPV-negative cervical cancer-derived cell lines. Even though we observed a very significant difference between SiHa and Ca Ski PIR transcripts (figure 2*e*), this difference decreased when we analysed pirin at protein level (figure 2*f*). Since many regulatory mechanisms are involved between transcription and translation, we cannot deny the possibility that such regulatory mechanisms may be differentially involved in SiHa and Ca Ski cells. For instance, differential epigenetic mechanisms (i.e. microRNAs) or differential factors influencing pirin stability might explain these results. In addition, here we showed that pirin upregulation depends on HR-HPV E6 and E7 oncoproteins in Ca Ski cells because when these oncoproteins are silenced, a significant decrease in PIR transcripts was observed. Moreover, we demonstrated that HR-HPV E7 plays a key role promoting PIR overexpression. This is the first study reporting HR-HPV-dependent PIR upregulation in oral and cervical cancer cells. Pirin, the product of the PIR gene, is a small protein composed of 290 amino acids with a molecular mass of 32 kDa [17]. It has been demonstrated that pirin is a non-haeme iron-binding protein that belongs to the cupin superfamily [18]. Although PIR is widely expressed in normal and tumour tissues, its function is poorly known. Only recently it was reported that pirin suppresses E-cadherin expression and is involved in the regulation of Snail and Twist expression in HeLa cells, suggesting that pirin is involved in EMT and regulation of cancer metastasis [19].

Our results suggest a new pathway for EMT promoted by HPV E6 and E7 oncoproteins that is mediated by pirin overexpression. It is widely known that E6 and E7 oncoproteins are involved in EMT directly by downregulating E-cadherin and upregulating vimentin, as well as through transcription factors as Snail, Zeb and Twist [20,21]. In addition, these oncoproteins indirectly regulate EMT through Jagged1 or via FGF2/4 through the PI3 K pathway [22]. However, the mechanism by which pirin mediates EMT is only partially known. In fact, it was reported that a pirin mutant which was defective for Bcl3 binding was able to decrease the levels of E-cadherin, suggesting that pirin induces EMT independently of Bcl3-Slug signalling [19].

Although our findings suggest specific HPV-dependent PIR overexpression, alterations in PIR expression have also been observed in various tumours under conditions of oxidative stress, especially in tobacco smoke-associated cancers [23,24]. In this respect, it was previously reported that pirin protein is strongly related to oxidative stress response. Under non-oxidizing conditions, it is considered that this protein resides in the nucleus. However, in some specific conditions related to an increased oxidative stress such as tobacco smoke exposition, activated pirin by oxidized iron-binding works as a positive regulator of NF-κB, which is a family of transcription factors involved in cell growth, survival and immune response that are constitutively activated in cancer [25]. It is now clear that HR-HPV E6/E7 oncoproteins modulate NF-κB signalling although remains controversial whether these oncoproteins activate or repress this transcription factor. However, it also appears that NF-κB activation or repression is tissue-dependent [26]. Understanding the relationship between HPV and NF-κB activation is crucial, and our findings suggest the existence of a new mechanism mediated by PIR for HR-HPV-dependent NF-κB activation.

As HR-HPV E7 lacks DNA binding activity, the possibility of a direct PIR promoter activation is unlikely, although we cannot discard the possibility that such activation may occur through some E7 interactions and formation of complexes allowing an increased activity of the PIR promoter. Previously, it was demonstrated that upon MEK1 inhibition, a significant decrease or pirin protein levels was observed in C-JUN transformed human fibroblasts [27]. Here, using specific signalling pathways inhibitors, we demonstrated that EGFR/MEK/ERK and probably PI3 K/AKT signalling pathways are important for E7-mediated PIR overexpression in oral cells (figure 4*a*,*b*). Thus, these findings suggest an interplay between MAPK and NF-κB signalling pathways during HPV-mediated carcinogenesis in which pirin would be an important mediator. Previously, it was reported that both HPV-16 E6 and E7 oncoproteins promote an increase in EGFR transcripts in human keratinocytes [28], in agreement with the importance of EGFR for E7-dependent PIR upregulation.

Even though HR-HPV E6 and E7 oncoproteins efficiently immortalize epithelial cells, additional alterations are required for complete cell transformation and tumour establishment [29,30]. We previously reported an interplay between tobacco smoke and HR-HPV E6 and E7 oncoproteins that increases proliferation and tumour properties in a model of lung epithelial cells [13]. As it was reported that PIR is upregulated in bronchial cells after tobacco smoke exposition [23], it is plausible that pirin may play a role in this process. On the other hand, we previously reported an increase in single- and double-strand DNA breaks in lung cells expressing ectopically HPV-16 E6 and E7 oncoproteins exposed to cigarette smoke condensate. Moreover, we observed an increase in γ-H2AX phosphorylation [14]. Considering the results obtained in this study, it is plausible that an increase in DNA damage and activation of the DNA damage response (DDR) in the presence of HR-HPV is related to pirin increase in oral or cervical cancer cells. Additional studies are necessary to test this possibility.

To our knowledge, the overexpression of PIR has not been previously reported in transcriptomic or proteomic studies of oral or cervical carcinomas. A recent report analysed 30 primary HNCs in order to identify differentially expressed genes between HPV-16-positive and HPV-negative samples. Results indicated that pirin was not overexpressed in HPV-positive tumours [31]. Another study reported up- and downregulated genes in HNCs stratified by HPV presence. Some signalling pathways such as those regulated by NF-κB were found altered, although PIR was not identified [32]. As HR-HPV and tobacco smoke-associated head and neck cancers are different clinical entities, an explanation for these findings is that PIR expression may not be significantly different between tobacco smoke- and HPV-associated HNCs.

**Figure 6.**
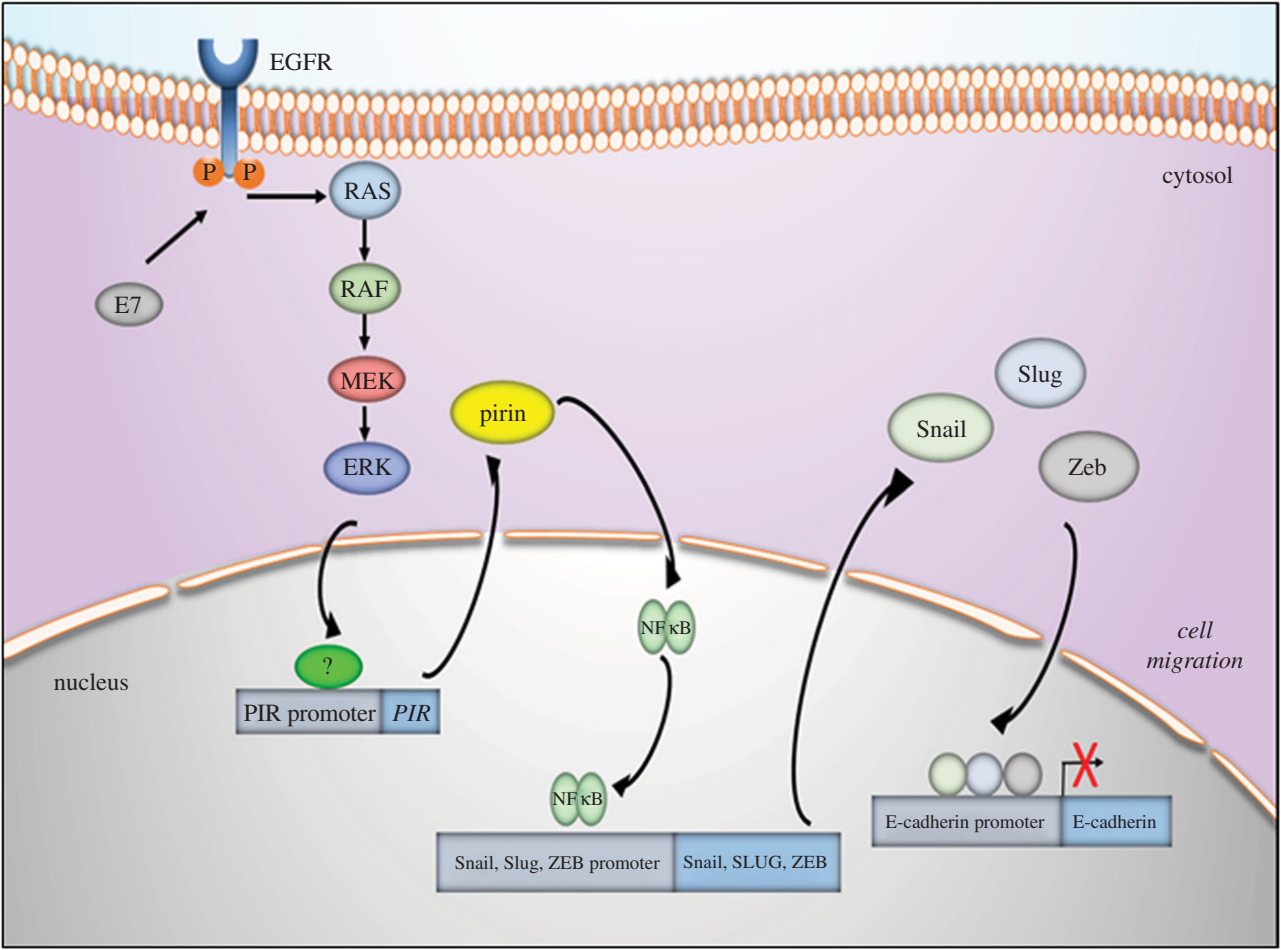
A proposed model for experimental data found in this study.

Taken together, our results show that HPV-16 E6/E7 induce pirin expression in oral and cervical cancer cells and that this effect is associated with the expression of EMT markers. These observations suggest that HPV oncogene-mediated pirin overexpression may be important in tumour progression inducing EMT in oral and cervix–uterine cancer cells. Considering the findings of this study, a proposed model is shown in figure 6. The potential value of pirin expression as a surrogate biomarker of HR-HPV in head and neck or cervical cancer warrants additional investigation.

## 4. Material and methods

### 4.1. Cell lines, plasmids, transfections and inhibitors

SiHa (HTB-35) and Ca Ski (CRL-1550) cell lines were obtained directly from the American Type Culture collection (ATTC, Manassas, VA, USA). C33A and C4I cells were kindly donated by Dr Priscilla Brevi, La Frontera University, Temuco, Chile. The cells were incubated in RPMI1640 basal medium (Gibco, Carlsbad, CA, USA) supplemented with 10% fetal bovine serum (FBS) (Hyclone, Fremont, CA, USA) with antibiotics (gentamicin, penicillin and streptomycin) and maintained at 37°C with 5% CO_2_ atmosphere. OKF6–Tert2 cells were kindly donated by Dr Denisse Bravo from Faculty of Odontology, University of Chile, and the cells were maintained in KSFM medium (Gibco) and DMEM high glucose (Gibco), respectively. For subculture, the cells were incubated with trypsin for 3–5 min and maintained with new medium containing FBS (Hyclone). The cells were periodically tested for mycoplasma contamination. PLXSNHPV16E6, pLXSNHPV16E7 and pLXSNHPV16E6E7 recombinant vectors were kindly donated by Dr Massimo Tommasino from International Agency for Research on Cancer (IARC), Lyon, France. The OKF6–Tert2 cells were stably transfected with pLXSN plasmids using lipofectamine 2000 (Invitrogen, Carlsbad, CA, USA) according to the manufacturer's instructions and were then selected using G418. For inhibition experiments, OKF6–Tert2 cells were transiently transfected with pLXSNHPV16E7 vector during 18 h. The cells were serum-depleted during 24 h and incubated with 4.6 µM AG1478 (Sigma, St Louis, USA), 6.3 µM UO126 (Sigma) or 10 µM LY294002 (Sigma) during 3 h. Then, the cells were washed twice with phosphate-buffered saline (PBS) (pH 7.4), dried and fixed for 5 min with TREVORS solution for IF assays.

### 4.2. Expression microarrays

Fifteen micrograms of RNA of stably OKF6–Tert2 transfected cells with an empty pLXSN or pLXSNE6E7 vector were labelled by reverse transcription in a 35 µl reaction containing 3 µg random primers, 3 µg oligo-dT, 10 nmol dATP, dGTP and dTCP, 4 nmol TTP, 2.5 nmol of either Cy3 or Cy5 of dUTP, 40 U RNAsin and 800 U MMLV reverse transcriptase (Promega, Madison, WI, USA). After incubation at 37°C for 2 h, 300 U of enzyme were added and further incubated for 2 h. The reaction was stopped with EDTA and residual RNA was hydrolysed with NaOH at 65°C for 20 min. The NaOH excess was neutralized by addition of 3 M sodium acetate, pH 5.2. The labelled cDNA was purified with MinElute columns (Qiagen, Hilden, Germany). Absorbance readings at 260, 550 and 650 nm were measured to estimate labelling efficiency using a Nanodrop1000 spectrophotometer (Thermo Scientific). Thirty-nine microlitres of labelled cDNA were combined with 45 µl 2× formamide solution (0.19% SDS, 0.196 µg ml^−1^ fish sperm DNA, 10× SSC buffer) and incubated at 95°C for 3 min. Then 90 µl of this solution was deposited onto 48 K-HEEBO oligonucleotide microarrays (Microarrays Inc., AL, USA) and mounted inside hybridization chambers (ArrayIt, CA, USA) for 22 h at 42°C. The slides were washed in four SSC, SDS-based solutions of decreasing stringency for 2 min each, dried by low-speed centrifugation and then scanned in a ScanArray Lite instrument (Perkin Elmer, MA, USA). The images obtained by scanning the arrays were quantified with the GenePix Pro 6.0 software, using the HS-Human-MI-ReadyArray*gal* file. Then, data were normalized with the online DNMAD software (http://dnmad.iib.uam.es/). The statistical analysis to determine differential expression was performed with a SAM test within TMEV (MultiExperiment Viewer) software, using a Delta value = 1. Finally, the pathway analyses were based in Gene Ontology and KEEG, and were performed with the online tool WebGestalt (WEB-based Gene Set Analysis Toolkit).

### 4.3. Western blot

Total protein was extracted from cells with a lysis buffer (100 mmol l^−1^ Tris pH 8.0, 1% SDS) containing both protease and phosphatase inhibitor cocktail (Roche, Basel, Switzerland). The cells were incubated at 4°C for 1 h, sonicated on ice for 20 s and centrifuged at 12 000*g* for 10 min. The proteins were quantified using the Pierce BSA Protein assay kit (Pierce, Rockford, IL, USA) and 30 µg of the extract was loaded per well. Following 12% PAGE–SDS, the proteins were transferred to a Hybond-P ECL membrane (Amersham, Piscataway, NJ, USA) using 20 mM Tris, 150 mM glycine pH 8.3, in 20% methanol with the semi-dry transfer apparatus (Bio-Rad). Membranes were incubated for 1 h at room temperature with the blocking reagent (5% bovine serum albumin, Tris buffered saline–0.5% Tween 20 pH 7.6), and incubated for 1 h at room temperature with the primary antibody against pirin, β-actin (Abcam, Cambridge, UK) diluted 1/1000 and 1/5000 in TBS–T20 (Tris-buffer Saline–Tween 20). Membranes were washed three times in TBS–T20 and incubated with secondary anti-IgG-labelled peroxidase (BD Pharmingen, San Diego, CA, USA). After washing three times in TBS–T20, immune complexes were detected using the ECL system (Amersham Pharmacia Biotech) according to the manufacturer's instructions.

### 4.4. Immunofluorescence and confocal microscopy

The cellular localization of expressed proteins was checked using IF. Transfected and non-transfected cells were grown to confluence in Chamber Slides, washed twice with PBS (pH 7.4), dried and incubated for 5 min with cold acetone. The fixed cells were then stored at −20°C until use. Cells were incubated with 1% bovine seroalbumin (BSA) for 30 min at room temperature, followed by incubation with a primary monoclonal anti-specific protein antibody diluted in PBS according to the manufacturer's instructions. The fixed cells were washed three times for 5 min at room temperature and incubated with a secondary FITC-labelled anti-IgG antibody. After three washes with PBS, the cells were visualized in a C2 Plus confocal microscope.

### 4.5. Reverse transcriptase–quantitative polymerase chain reaction

The RNA from cell lines was isolated using Trizol reagent (Invitrogen) according to the manufacturer's instructions. After chloroform purification and isopropanol precipitation, the RNA was suspended in DEPC-water and stored at −80°C. The RNA was treated with RQ1 RNase-free DNase (Promega) at 37°C for 60 min and then incubated with RQ1 DNase Stop Solution for 10 min. The cDNA was prepared using a 20 µl reaction volume containing DNAse-treated RNA (2 µg), 1 U RNAse inhibitor (Promega), 0.04 µg µl^−1^ random primers (Promega), 2 mM dNTP (Promega) and 10 U Moloney Murine Leukemia Virus (MMLV) reverse transcriptase (Promega). The reaction mixture was incubated for 1 h at 37°C. The cDNA was subjected to real-time PCR quantification of gene expression with specific primers (electronic supplementary material, table S1) in a Rotor-Gene 6000 system (Corbett Research, Sydney, Australia) under the following conditions: 95°C for 10 min followed by 40 cycles of denaturation at 95°C for 15 s, annealing at 55°C for 20 s and extension at 72°C for 20 s. The condition for the dissociation curve was increasing temperature from 70 to 90°C, 0.5°C at each step. The reaction was performed using 2 × SYBR Green Master Mix (Bioline, London, UK), 0.4 µM primer pair, 10.5 µl RNase-free water and 1 µl cDNA in a 25 µl final volume. Specific primers and amplification conditions were adjusted for each RNA sequence. Standard curves for each gene were generated independently by preparing 10-fold serial dilutions of DNA amplicons. The relative copy number of each sample was calculated through of 2(−ΔΔ*C*(T)) method using Rotor-Gene software. Endogenous β-actin mRNA levels were used for normalization of RNA expression. All reactions were performed in duplicate.

### 4.6. Migration assays

For the three-dimensional migration assay, the bottom side of a transwell upper chamber (Corning, Mexico) was coated with 2 µg ml^−1^ fibronectin and incubated overnight at 4°C. A total of 30 000 Ca Ski or SiHa cells were seeded in non-supplemented medium inside the upper chamber, and 500 µl of complete medium was added to the plate. Cells were allowed to migrate for 7 h. Migrated cells were fixed and stained with crystal violet/MetOH solution. Non-migrated cells were removed with a cotton tip. Migrated cells were counted in eight fields for each experiment.

### 4.7. Statistical analysis

The comparison of means was made using Student *t*-test with Stata 11 software. *p*-values of less than 0.05 were considered statistically significant.

## Supplementary Material

HPV-16 E6 and E7 transcripts are expressed and are functional in oral OKF6 cells stably transfected with pLXSNHPV-16E6/E7 vector

## Supplementary Material

OKF6 cells expressing HPV-16 E6 and E7 oncoproteins show increased proliferation

## Supplementary Material

HPV-16 E6 and E7 oncoproteins are functional in oral OKF6 cells

## Supplementary Material

siRNAs for E6 and E7 silencing are functional

## Supplementary Material

siRNA for PIR silencing is functional

## Supplementary Material

The EGFR/MEK/ERK and PI3K/AKT pathways abrogation do not affect basal pirin levels in OKF6-Tert2

## Supplementary Material

PIR expression is associated to an increased migration in SiHa cells

## Supplementary Material

Supplementary Tables and Figures

## Data accessibility

The complete microarray dataset was deposited to GEO (https://www.ncbi.nlm.nih.gov/geo/) and made freely available through the accession code GSE102821 under the title ‘OKF6/Tert2 Human oral cells: control versus transfected with E6E7 HPV16’. Supplementary data are available at the journal's website.
