# Supplementary figures and images for: Upregulation of PIR gene expression induced by human papillomavirus E6 and E7 in epithelial oral and cervical cells
Source: Open Biol. 2017 Nov 8;7(11):170111. doi: 10.1098/rsob.170111 (PMC5717337; doi:10.1098/rsob.170111)

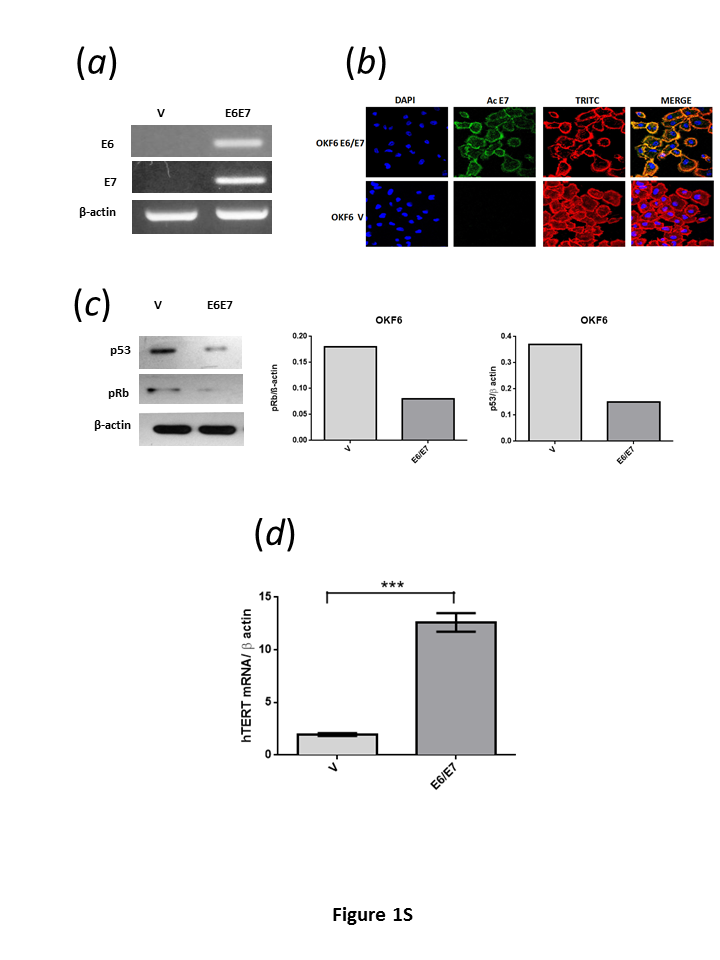

Supplement: HPV-16 E6 and E7 transcripts are expressed and are functional in oral OKF6 cells stably transfected with pLXSNHPV-16E6/E7 vector [file rsob170111supp1.tif]

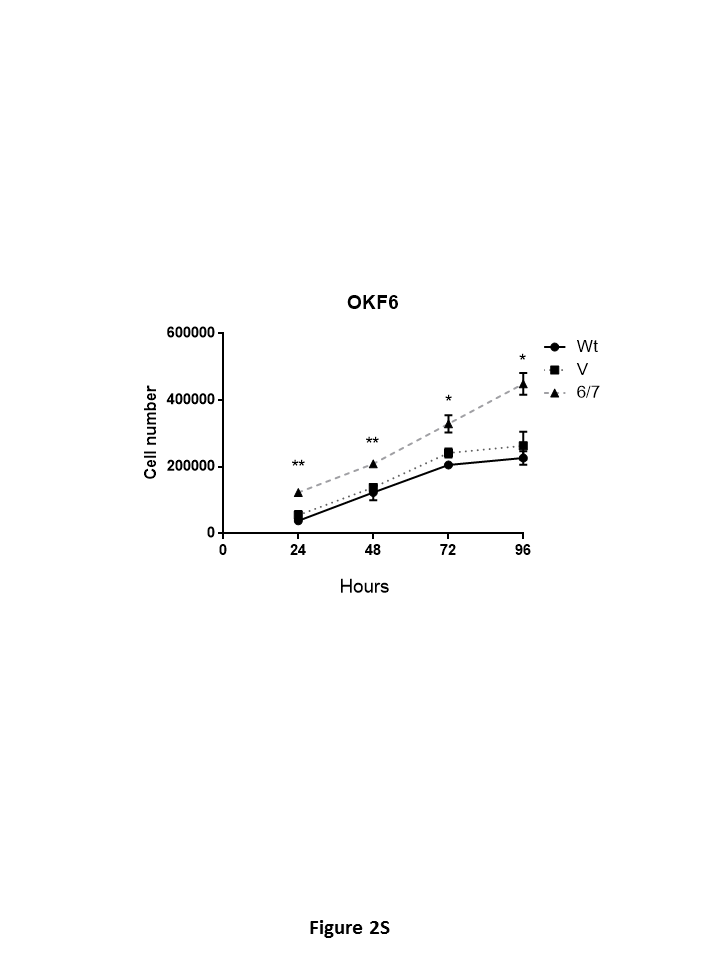

Supplement: OKF6 cells expressing HPV-16 E6 and E7 oncoproteins show increased proliferation [file rsob170111supp2.tif]

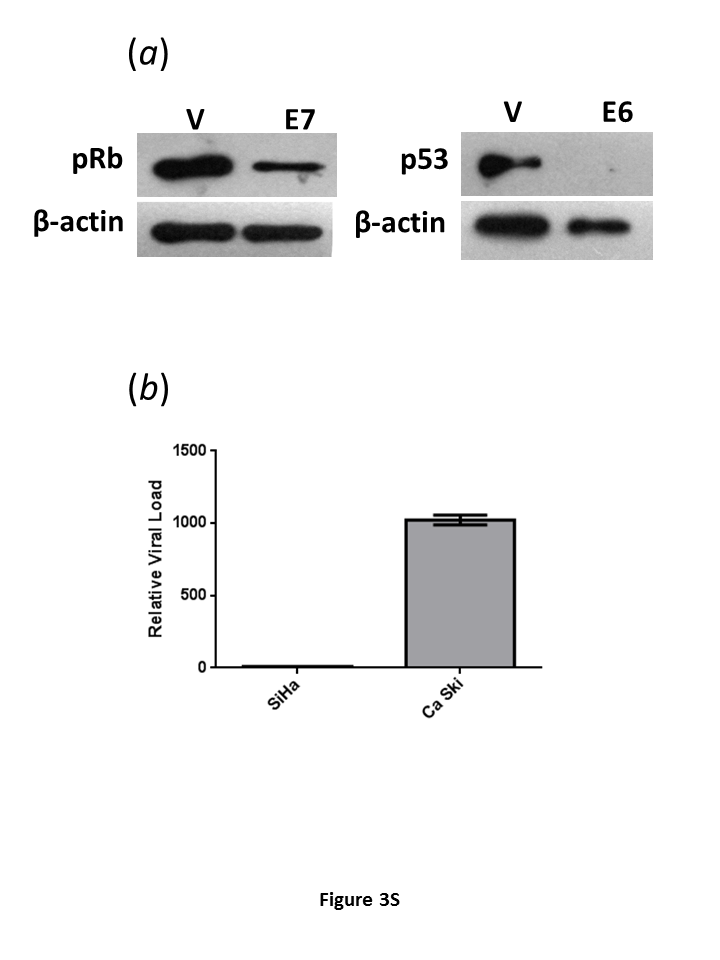

Supplement: HPV-16 E6 and E7 oncoproteins are functional in oral OKF6 cells [file rsob170111supp3.tif]

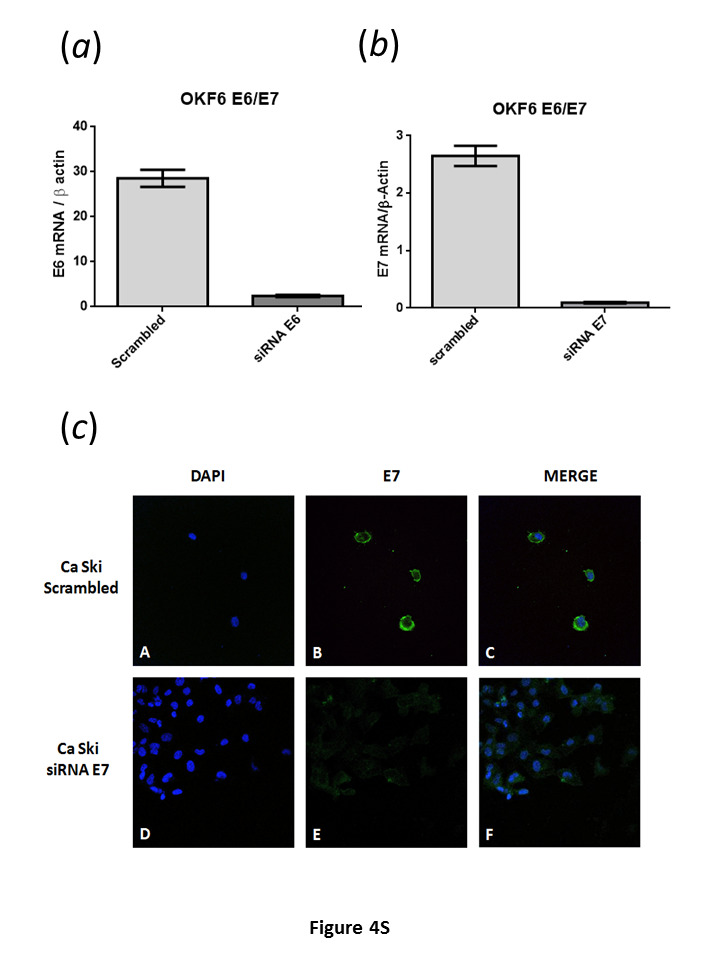

Supplement: siRNAs for E6 and E7 silencing are functional [file rsob170111supp4.tif]

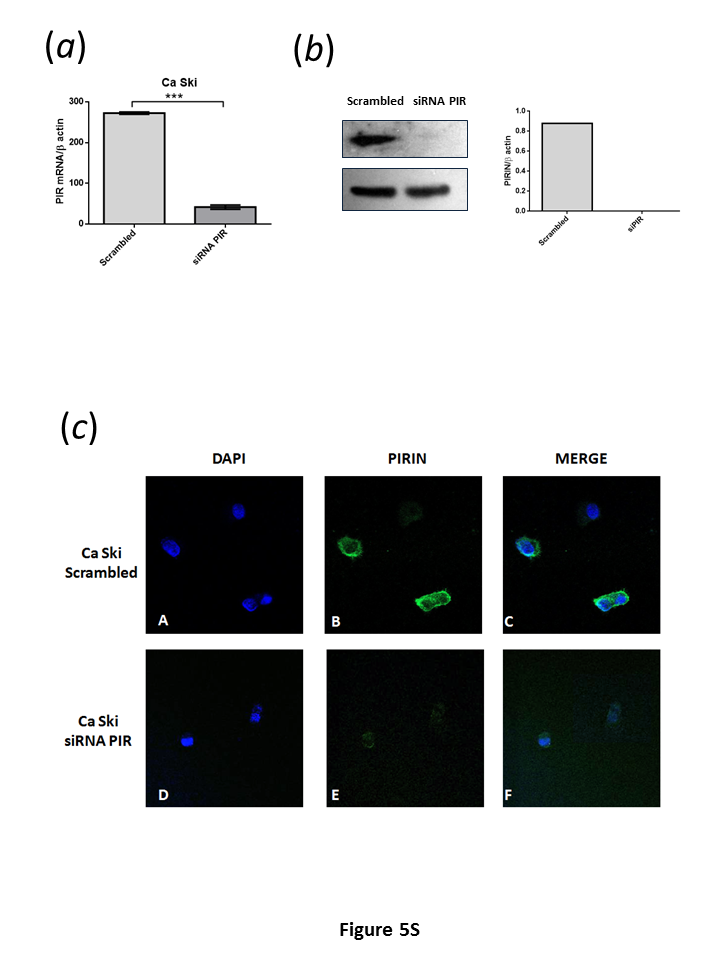

Supplement: siRNA for PIR silencing is functional [file rsob170111supp5.tif]

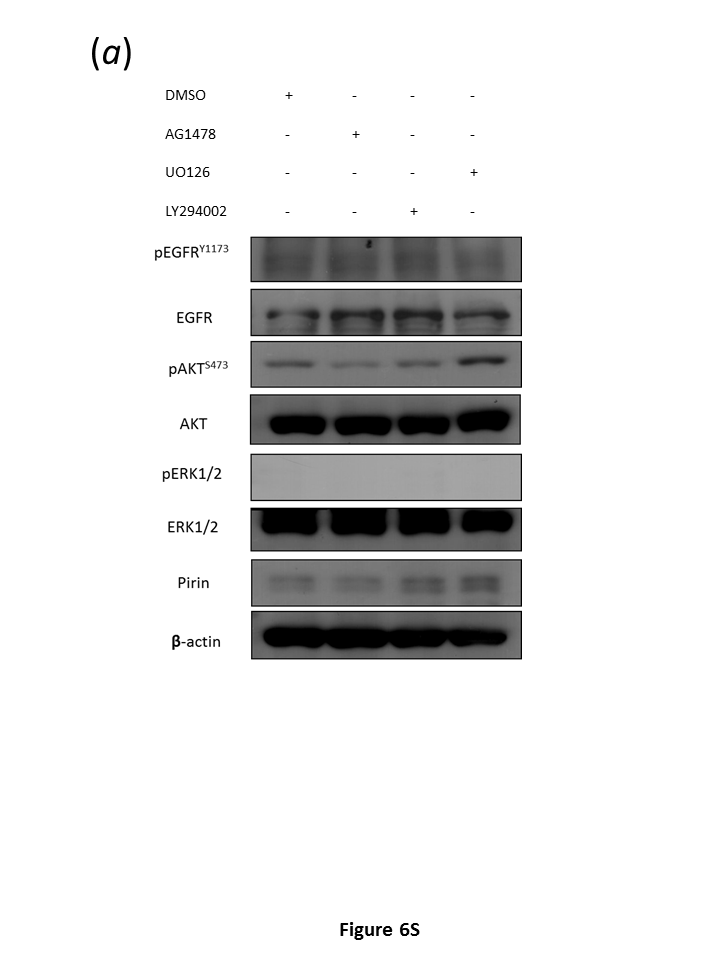

Supplement: The EGFR/MEK/ERK and PI3K/AKT pathways abrogation do not affect basal pirin levels in OKF6-Tert2 [file rsob170111supp6.tif]

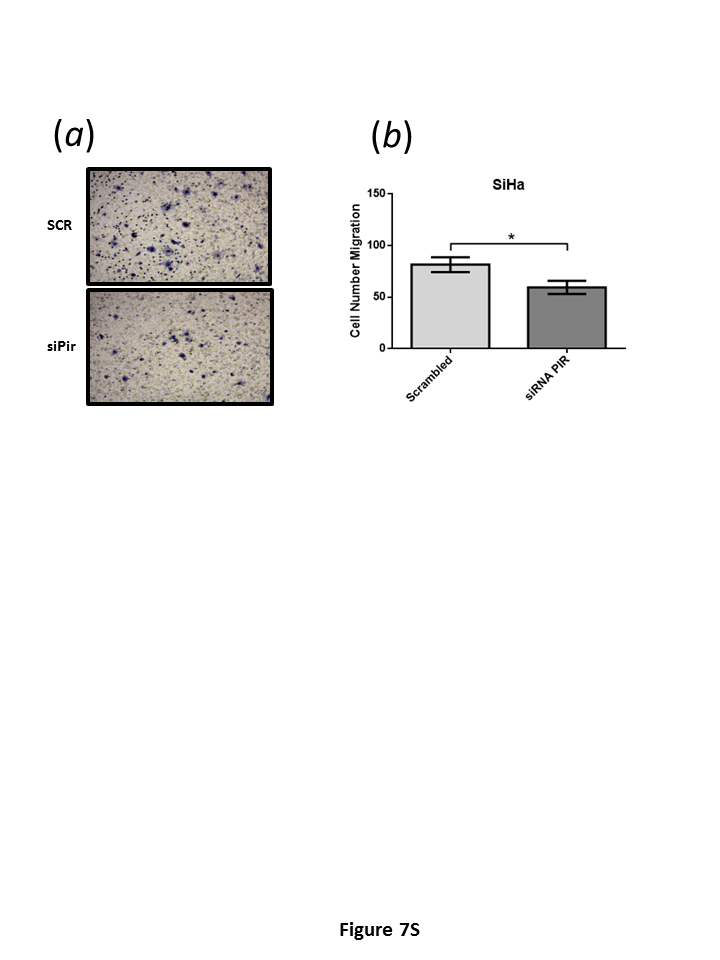

Supplement: PIR expression is associated to an increased migration in SiHa cells [file rsob170111supp7.tif]
