# Supplementary material for: Upregulation of PIR gene expression induced by human papillomavirus E6 and E7 in epithelial oral and cervical cells
Source: Open Biol. 2017 Nov 8;7(11):170111. doi: 10.1098/rsob.170111 (PMC5717337; doi:10.1098/rsob.170111)
Supplement: Supplementary Tables and Figures [file rsob170111supp8.docx]

**SUPPLEMENTARY TABLES AND FIGURES**

**Figure 1S.** HPV-16 E6 and E7 transcripts are expressed and are functional in oral OKF6-Tert2 cells stably transfected with pLXSNHPV-16E6/E7 vector. (*a*) Reverse transcriptase PCR for HPV-16 E6, E7 and β -actin transcripts in OKF6-Tert2 cells. (*b*) Immunofluorescence by confocal microscopy revealing E7 expression in OKF6-Tert2 cells. (*c)* Left side: Western blot for p53 and pRb proteins in OKF6-Tert2 cells. Right side: densitometric analysis. (*d, e*) Quantitative transcriptase-reverse PCR for p16 and hTERT transcripts in OKF6-Tert2 cells that express HPV-16 E6 and E7. These results are representative of three independent experiments. ***: p<0.001.

**Figure 2S**. OKF6-Tert2 cells expressing HPV-16 E6 and E7 oncoproteins show increased proliferation. Oral OKF6-Tert2 cells were stably transfected with pLXSNHPV16E6E7 or pLXSN empty vector and cell viability was evaluated at different times (0 to 96 h) using MTS assays. These results are representative of three independent experiments.

**Figure 3S.** HPV-16 E6 and E7 oncoproteins are functional in oral OKF6-Tert2 cells. (*a*) OKF6-Tert2 cells were transiently transfected with pLXSNHPV16E6 (right), pLXSNHPV16E7 (left) or empty pLXSN vector and p53 or pRb proteins were evaluated through Western blot. Results are representative of three independent experiments. (*b*) Relative viral load in SiHa and Ca Ski cells determined by qPCR.

**Figure 4S.** siRNAs for E6 and E7 silencing are functional. (*a, b*) The usefulness of siRNAs and scrambled for HPV-16 E6 and E7 silencing was evaluated by RT-qPCR. (*c*) Immunofluorescence by confocal microscopy for HPV-16 E7 oncoprotein in Ca Ski cells transiently transfected with siRNA against E7 or scrambled. Results are representative of three independent experiments.

**Figure 5S.** siRNA for PIR silencing is functional. Ca Ski cells were transiently transfected with a siRNA or scrambled for PIR silencing and the levels of PIR transcripts or pirin protein were evaluated by (*a*) RT-qPCR, (*b*) Western blotting and (*c*) immunofluorescence. Results are representative of three independent experiments.

**Figure 6S.** The EGFR/MEK/ERK and PI3K/AKT pathways abrogation do not affect basal pirin levels in OKF6-Tert2**.** (*a*) OKF6-Tert2 cells stably transfected with pLXSN vector were incubated with inhibitors AG1478 (EGFR tyrosine kinase), UO126 (MEK1/MEK2) and LY294002 (phosphoinositide 3-kinase). Pirin protein levels were evaluated by Western blot.

**Figure 7S.** PIR expression is associated to an increased migration in SiHa cells. (*a*) Transwell assay for SiHa cell migration after transfection with a siRNA for PIR silencing, (*b*) Cell count after 7 h of migration. These results are representative of three independent experiments. *: p<0.05; **: p<0.01; ***: p<0.001.

**Table 1S.** Primers used in this study.

| Name | Primer forward | Primer reverse | Size |
| --- | --- | --- | --- |
| PIR | TCAAATTGGACCCAGGAG | TCCAAGCACTGCTGTGTG | 131 |
| E7 | ATTTGCAACCAGAGACAACTG | CAATATTGTAATGGGCTCTGT | 110 |
| E6 | CTGCAAGCAACAGTTACTGCG | TCACACACTGCATATGGATTC | 96 |
| ß-actin | AGCGAGCATCCCCCAAAG | GGGCACGAAGGCTCATCA | 285 |
| P16 | GGGCACCAGAGGCAGTAAC | TTCCCGAGGTTTCTCAGAGC | 100 |
| hTERT | GCGGAAGACAGTGGTGAACT | ACCTGGAGTAGTCGCTCTGC | 146 |
| E-cadherin | GCACCGGTCGACAAAGGACA | AGTCCCAGGCGTAGACCAAGA | 135 |
| ZEB-1 | ACTGCCTGGTGATGCTGAAA | CCCAAACTGCAAGAAACGCT | 157 |
| Slug | CTCCATTCCACGCCCAGCTAC | AGCCACTGTGGTCCTTGGAG | 117 |
| Snail | AGGCTCGAAAGGCCTTCAACT | TGTGGCTTCGGATGTGCATCT | 84 |
